# *In silico* Approach for Anti-Thrombosis Drug Discovery: P2Y_1_R Structure-Based TCMs Screening

**DOI:** 10.3389/fphar.2016.00531

**Published:** 2017-01-09

**Authors:** Fan Yi, Le Sun, Li-jia Xu, Yong Peng, Hai-bo Liu, Chun-nian He, Pei-gen Xiao

**Affiliations:** ^1^Institute of Medicinal Plant Development, Peking Union Medical College, Chinese Academy of Medical SciencesBeijing, China; ^2^Key Laboratory of Bioactive Substances and Resources Utilization of Chinese Herbal Medicine, Ministry of EducationBeijing, China

**Keywords:** Traditional Chinese medicines, P2Y_1_R, anti-thrombosis, platelet aggregation, *in silico* screening

## Abstract

Cardiovascular diseases (CVDs), including thrombosis, which is induced by platelet aggregation, are the leading cause of mortality worldwide. The P2Y_1_ receptor (P2Y_1_R) facilitates platelet aggregation and is thus an important potential anti-thrombotic drug target. The P2Y_1_R protein structure contains a binding site for receptor antagonist MRS2500 within its seven-transmembrane bundle, which also provides suitable pockets for numerous other ligands to act as nucleotide antagonists of P2Y_1_R. The Traditional Chinese Medicine Systems Pharmacology Database and Analysis Platform (TCMSP) comprises 499 Chinese Pharmacopoeia-registered herbs and the structure information for 29,384 ingredients. *In silico* docking of these compounds into the P2Y_1_R protein structure within the MRS2500 pocket can identify potential antithrombotic drugs from natural medicinal plants. Docking studies were performed and scored to evaluate ligand-binding affinities. In this study, a total of 8987 compounds from Traditional Chinese Medicine (TCM) were filtered by Lipinski's rule of five, and their ideal oral-intake properties were evaluated. Of these, 1656 compounds distributed in 443 herbs docked into the P2Y_1_R-MRS2500 structure in 16,317 poses. A total of 38 compounds were ranked with a DockScore above 70, and these may have significant potential for development into anti-thrombosis drugs. These computational results suggested that licorice (*Glycyrrhiza uralensis* Fisch), cimicifugae (*Cimicifuga foetida* L.), and ganoderma (*Ganoderma lucidum* Karst) and their chemical constituents, which have not previously been widely used for anti-thrombosis, may have unexpected effects on platelet aggregation. Moreover, two types of triterpene scaffolds summarized from 10 compounds were distributed in these three herbs and also docked into P2Y_1_R. These scaffold structures may be utilized for the development of drugs to inhibit platelet aggregation.

## Introduction

Cardiovascular disease (CVD) is the leading cause of mortality worldwide. CVD is multifactorial, and its risk factors include stroke, hypertension, arrhythmias, and thrombosis (Mozaffarian et al., 2016). Platelet aggregation-induced thrombosis obstructs blood circulation, playing a central role in acute, and chronic arterial vascular diseases (Radomski et al., 2005). Antiplatelet drugs decrease thrombus formation, and their estimated market is worth 24 billion United States dollars (USD).

G protein-coupled P2Y receptors belong to the nucleotide receptor G protein-coupled receptor (GPCR) family and have eight mammalian subtypes (P2Y_1, 2, 4, 6, 11-14_) (Kim et al., 2003). P2Y_1_ and P2Y_12_ belong to the human purinergic GPCRs and can be activated by adenosine 5′-diphosphate (ADP) to induce platelet activation (Gurbel et al., 2015). ADP is the first small-molecular weight platelet agonist, and its receptors, such as P2Y_1_ receptors, can couple to activated phospholipase C. The activation of serotonin receptor supplements signaling through the P2Y_1_ receptor, demonstrating that it is a specific antagonist able to block ADP-induced platelet aggregation (Jin and Kunapuli, 1998). The human P2Y_1_ receptor protein structure and its two ligand-binding sites for the nucleotide-like antagonist MRS2500 and allosteric antagonist 1-(2-(2-tert-butylphenoxy)pyridin-3-yl)-3-(4-(trifluoromethoxy)phenyl)urea (BPTU) were reported in 2015 (Protein Database [PDB] ID: 4XNW, 4XNV) (Zhang et al., 2015). (1′R,2′S,4′S,5′S)-4-(2-Iodo-6-methylaminopurin-9-yl)-1-[(phosphato) methyl]-2(phosphato)bicycle[3.1.0]-hexane (MRS2500) is a reported antagonist candidate that exerts its effect via its unique chemical structure. This compound binds the recombinant human P2Y_1_ receptor and inhibits the platelet aggregation caused by ADP with an 50% inhibitory concentration (IC50) value in the nanomolar range. It also effectively reduces arterial thrombosis and prolongs bleeding time and has been evaluated as a prototypical antithrombotic agent both *ex vivo* and *in vivo* (Hechler et al., 2006). Unlike P2Y_12_R, P2Y_1_R has a highly conserved in class A GPCR residue P229. The pocket for MRS2500 binding to P2Y_1_R mainly defined by residues from the N terminus, ECL2, and its helices structures. In P2Y_1_R, the antagonist MRS2500 potentially prevents the movements of these helices and stabilized the receptor in an inactive state by interacting with helices In the P2Y_1_R–MRS2500 structure, each terminal oxygen of the two phosphates forms at least one contact with the receptor. The hydrogen bonds from 3′-phosphate with Arg195 and Thr201, meanwhile, it is engaged in two salt-bridge interactions with Lys46 at the N terminus. The 5′-phosphate forms a salt-bridge with Thr205 and makes hydrogen bonds with Asp204 and Arg310. P2Y_1_R and P2Y_12_R structures reveal very different features in binding their nucleotide-like ligands even though recognized by the same endogenous ligand ADP. Most significantly, the binding site of MRS2500 in P2Y_1_R locates much closer to the extracellular surface than the other known GPCR structures affiliated small-molecule ligand-binding sites. Due to its more safety advantage over the P2Y_1_R inhibitors of reducing bleeding liabilities than P2Y_12_R, it has been suggested to discovery as a whole new drug targets (Gachet, 2008). Moreover, the P2Y1R is also enrolled in other procedure in human body, such as activation of extracellular signal-regulated kinase in astrocytes and vascular inflammation (Zerr et al., 2011). In recent years, many study were performed on P2Y_12_R, however, the P2Y_1_R protein and its ligand pocket crystal structure was firstly reported in 2015. And P2Y_1_R has the specificity bind-model and its diversity of regulation mechanisms compared with P2Y_12_R. This give us a hit that it is easier for other unknown small-molecule can binding to the pocket and thus be a potential antagonist of P2Y_1_R.

Traditional Chinese Medicines (TCMs) is a relevant source of biological compounds with innovative mechanisms of action. Although TCMs concepts diverge from Western medicine, concepts, some correlations exist between the two systems. For example, *Huoxuehuayu* promotes blood circulation and alleviates blood stasis. Several compounds, including ligustrazine, saffionin A, resveratrol, propylgallate, notoginseng triterpenes, crudione, lerulic acid, salvianolic acid A and B, tanshinone IIA, and safflower flavin, which are extracted from the TCM herbs: *Salvia miltiorrhiza* Bge., *Rheum palmatum* L., and *Ligusticum chuanxiong* hort., can inhibit platelet aggregation induced by arachidonic acid (AA), ADP, platelet-activating factor (PAF) and collagen or thrombin both *in vivo* and *in vitro* (Chen, 1981). Moreover, because the mechanism of thrombosis involves multiple pathways and requires a multi-target approach for treatment, various small molecule compounds that are not limited to the TCMs activity for the treatment of platelet aggregation can be used to target different proteins, tissues and organs. Therefore, other TCM plants and their active ingredients may act on the P2Y_1_ receptor protein to inhibit platelet aggregation via an innovative approach.

Computational approaches have been widely applied to molecular biology and medicinal research. Structure-based methods, such as ligand-protein docking, are efficient and reliable tools for novel drug discovery and design. *In silico* docking can elucidate the interactions and binding mechanisms between the protein target and its suitable ligands. The Traditional Chinese Medicine Systems Pharmacology Database and Analysis Platform (TCMSP) comprises all 499 Chinese herbs registered in the Chinese Pharmacopoeia and 29,384 ingredients (Ru et al., 2014). The TCMSP provides a total of twelve absorption, distribution, metabolism, and excretion (ADME)-related properties, including oral bioavailability and half-life.

The objective of this study was to use an *in silico* docking approach to investigate the potential of compounds in the TCMSP and their distributed herbs as P2Y_1_R antagonists and potential new antiplatelet drugs. In this study, we collected all the information of herbs and their contained ingredients from the TCMSP. Then performing the docking procedure of these total 29,384 compounds into the pocket structure of receptor P2Y_1_R. Analyzed results of these high efficient docked compounds, summarized there general characters such as scaffold and there distributed herbs will provide a new direction in antiplatelet drugs innovation.

## Materials and methods

### Software

The 2-dimensional (2D) and 3D structures of TCM compounds were drawn using ChemBioOffice 2010 (PerkinElmer Inc., Cambridge, MA, USA) and MarvinSketch V15.8 (ChemAxon Ltd., Budapest, Hungary). The P2Y_1_ protein and MRS2500 structure (PDB ID: 4XNW) were downloaded from the PDB database (http://www.rcsb.org/pdb) and optimized with Molecular Operating Environment (MOE) 2014.09 (Chemical Computing Group Inc., Montreal, QC, Canada). Candidate compounds were downloaded from the TCMSP database (http://lsp.nwsuaf.edu.cn/tcmsp.php). The chemical structure format was transformed by OpenBabelGUI (O'Boyle et al., 2011) version 2.3.2 (OpenBableGUI 2006 by Chris Morley). Virtual screening was performed using Discovery Studio Client V4.5 (DS 4.5; Accelrys Inc., San Diego, CA, USA). The network and functional analyses were generated using QIAGEN's Ingenuity Pathway Analysis (IPA®, QIAGEN Redwood City, CA, USA; http://www.qiagen.com/ingenuity).

### Lipinski's rule of five and ADME/toxicity (T) predictions

Those compounds collected in the TCMSP database were evaluated for Lipinski's rule of five (RO5; Lipinski et al., 2012) before screening. Poor pharmacokinetics and toxicity are important causes of costly failures during the downstream process of drug development. This evaluation procedure was performed in DS 4.5, the “filter by Lipinski and Veber Rules” which located in “Small Molecules” module. All setting as default values which individual property thresholds according to the original publications. Their ADME/T properties (van de Waterbeemd and Gifford, 2003) must be predicted and are now computable with *in silico* approaches, accelerating the drug discovery process. Therefore, we can also filter and design drug-like molecules. The DS 4.5 ADME/T Description module was used to predict the pharmacokinetic properties of all candidate compounds. This program offers reliable predictions for pharmaceutically relevant properties, including aqueous solubility, blood-brain barrier penetration (BBP), cytochrome P450 2D6 inhibition, hepatotoxicity, human intestinal absorption (HIA), and plasma protein binding. The ADME/T Description predicts the physically significant descriptors of pharmaceutically relevant properties of organic molecules. The BBP-95&99 and Absorption-95&99 confidence intervals are demonstrated as four elliptical rings in the ADME/T_AlogP98 & ADME/T_PSA_2D (Y–X axis) coordinate system.

### Docking analysis

The virtual screening docking process was performed with the LigandFit module. LigandFit gives the best poses at the binding site using a stochastic conformational search and the energy of the ligand-protein complex (Sato et al., 2006). Compounds from the TCMSP database were docked to the P2Y_1_ protein active site fit for MRS2500 reported by Zhang (Zhang et al., 2015). All procedures were completed using Chemistry at HARvard Molecular Mechanics (CHARMm; Brooks et al., 2009). The 3D multi-conformational compound molecular structures were generated using Monte Carlo algorithm-based conformational analysis and rigid body minimization completed using the Smart Minimizer module. The DockScore was adopted as the LigandFit scoring to rank the TCMSP compounds that docked into the P2Y_1_R ligand-binding pocket for MRS2500. The LigandFit docking procedure consists of the following two steps: (1) identify and select the region of the protein as the active site for docking by cavity detection and (2) dock the candidate ligands to the selected site. The docking cavity was defined using the DS site search module. For all potential drugs, the docking site was derived from the position of the MRS2500 and P2Y_1_ co-crystallized construction (PDB ID: 4XNW), and the grid resolution was set to 0.5 Å (default). The ligand-accessible grid was defined as the minimum distance between a grid point and the protein and was 2.0 Å for hydrogen and 2.5 Å for heavy atoms. This confirmed binding site was used to calculate the non-bonded interactions between all potential compounds and the P2Y_1_ receptor protein residues. The docking procedure was initiated with the generation of random ligand conformations. The following procedures were performed after a new conformation was generated: The shapes of the potential ligands were compared with the active site, and if the result was acceptable, the dock energy (DockScore) was computed between the protein and ligand trial conformation. Variable numbers of Monte Carlo steps were used to generate different ligand conformations. Scoring was performed with six scoring functions: DockScore, LigScore 1 and 2, Piecewise Linear Potential (PLP) 1 and 2, and Potential of Mean Force (PMF). We assumed that the bioactive orientations ranked by DockScore and other scoring functions were used to retain each molecule and compute the enrichment factors.

## Results and discussion

### Screening and structure analysis

**Figure 1:**
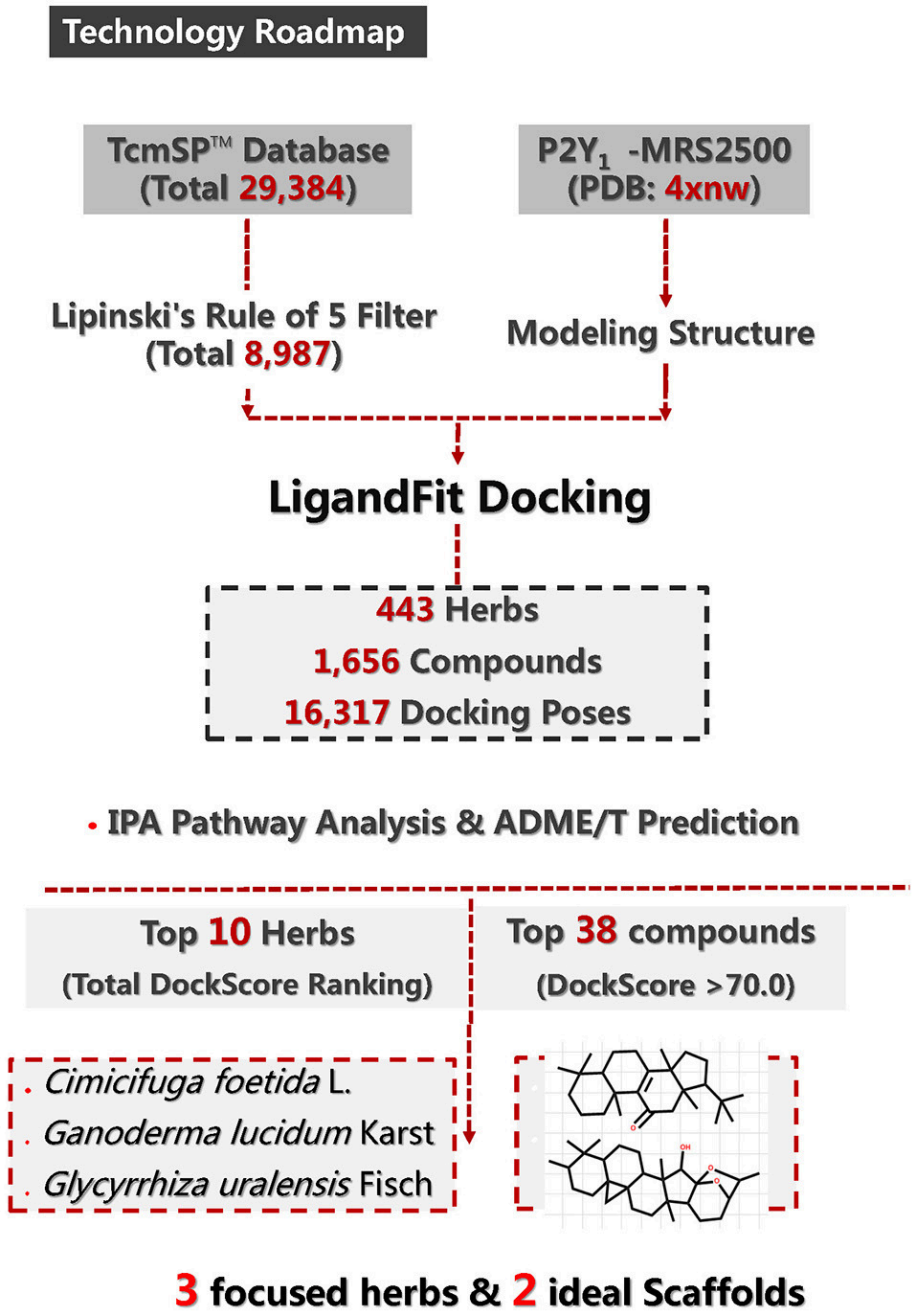
**Methodology roadmap and major results for P2Y_1_R-related anti-thrombosis drug design from the TCMSP database**.

**Figure 2:**
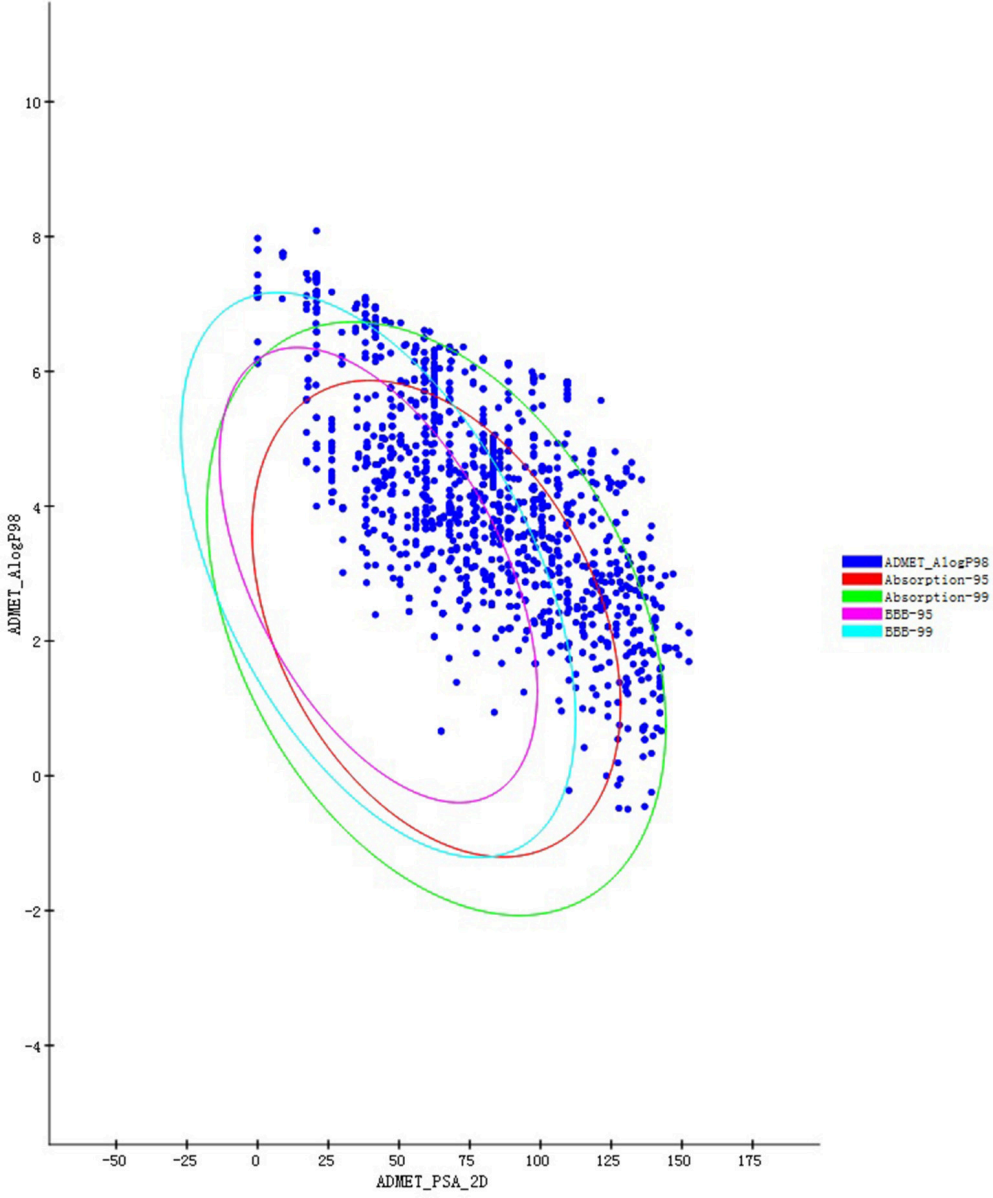
**ADME/T prediction plots for compounds that successfully docked in P2Y_1_R (1656) from among the compounds in the TCMSP database**.

Figure 1 summarizes the technology roadmap and the screening results after each step. In 1997, Lipinski led to the well-known “Rule of Five” (RO5) for selecting drug-like molecules, which demonstrated orally administered drugs should have good oral bioavailability in order to be effective. According to the “Rule of Five,” a drug-like molecule should have no more than one of the following criterias: No more than five hydrogen bond donors, no more than 10 hydrogen bond acceptors, a molecular mass <500 daltons, and an octanol-water partition coefficient log P not >5. To ensure this study with a reliable and available results. All compounds need to filtered by the RO5. From the 29,384 native TCM compounds collected from the TCMSP database, 8987 compounds were filtered by Lipinski's rule of five and prepared for further study. Successful docking to P2Y_1_R was observed for 1656 compounds (16,317 poses), and their ADME/T descriptors were calculated (Figure 2, Supplementary Datasheet 1). According to the DS 4.5 suggested theory, the Human Intestinal Absorption (HIA) model can embodied in ADMET plot the validate the predictions. The ADMET—Human Intestinal Absorption model predicts human intestinal absorption (HIA) after oral administration. Intestinal absorption is defined as a percentage absorbed rather than as a ratio of concentrations. A well-absorbed compound is one that is absorbed at least 90% into the bloodstream in humans. These levels are defined by the 95% (red line) and 99% (green line) confidence ellipsoids in Figure 2. For the ADME/T_PSA_2D, ADME/T_AlogP98 plane, two confidence ellipsoids of 95 and 99% for oral absorption surrounded most of these natural compounds. Two ellipsoids of 95 and 99% for BBP with high penetrations further refined the range of compounds suitable for clinical development as oral-intake drugs. Total view of these 1656 compounds, majority number within an acceptable range which indicated that most candidates compounds have an ideal human intestinal absorption which can further refined the range of compounds suitable for clinical development as oral-intake drugs. Candidate ligand poses can be evaluated and prioritized according to the DockScore function. Each docked compound has an optimal pose indicated by its highest DockScore. A total of 38 compounds had a DockScore above 70.0, and these presented high potential for further studies (Table 1).

**Table 1:** **Docking results of the top 38 (DockScore > 70) and their plant sources**.

**Molecule Name**	**Pubchem_CID**	**DockScore**	**TCMs**
Ganoderic acid J	20055991	78.772	***Ganoderma lucidum*** **Karst**
Cimiside E	399180	78.104	***Cimicifuga foetida*** **L**.
6″-O-Acetylliquiritin	101051311	77.347	***Glycyrrhiza uralensis*** **Fisch**
7,8-Didehydrocimigenol	101577840	76.088	***Cimicifuga foetida*** **L**.
(2S,6R)-6-[(5R,7S,10S,13R,14R,17R)-7-Hydroxy-4,4,10,13,14-pentamethyl-3,11,15-trioxo-1,2,5,6,7,12,16,17-octahydrocyclopenta[a]phenanthren-17-yl]-2-methyl-4-oxoheptanoic acid	10481601	75.198	***Ganoderma lucidum*** **Karst**
Cimicifoetiside A	16019999	74.951	***Cimicifuga foetida*** **L**.
2′,7-Dihydroxy-4′-methoxyisoflavan-7-O-β-d-glucopyranoside	/	74.906	***Glycyrrhiza uralensis*** **Fisch**
Lappaol C	323896	74.793	*Arctium lappa* L.
Diosgenin glucoside	65609	74.599	*Asparagus cochinchinensis* (Lour.) Merr.
2-(2-Phenylethyl)-6-[[(5S,6R,7R,8S)-5,6,7-trihydroxy-4-keto-2-(2-phenylethyl)-5,6,7,8-tetrahydrochromen-8-yl]oxy]chromone	14283395	74.349	*Sinapis alba* L.
Alisol C monoacetate	14036813	74.331	*Alisma orientale* (Samuels) Juzep.
Ganoderic acid E	23247894	74.216	***Ganoderma lucidum*** **Karst**
Ononin	442813	74.078	*Hedysarum multijugum* Maxim.
STOCK1N-49993	16401117	73.833	*Fritillaria cirrhosa* D. Don
Cimigenol	16020000	73.833	***Cimicifuga foetida*** **L**.
Lappadilactone	11081540	73.678	*Rosa banksiae* Ait.
Glyasperin E	392442	73.281	***Glycyrrhiza uralensis*** **Fisch**
Ganodermic acid Q	10436380	73.28	***Ganoderma lucidum*** **Karst**
Sophojaponicin	6326060	73.071	*Euchresta japonica* Hook. f. ex Regel *Rheum officinale* Baill.
Picroside III	24121289	72.681	*Neopicrorhiza scrophulariiflora* (Pennell) D.Y.Hong
Hirsutaside C	/	72.612	*Uncaria sinensis* (Oliv.) Havil.
Avenacoside A	71581001	72.551	*Lilium brownii var*. viridulum Baker
Fichotomide I	11244683	72.09	*Gypsophila licentiana* Hand.-Mazz.
Dioscoreside C_qt	52931427	71.807	*Dioscorea opposite* L.
Sanggenol A	15233693	71.63	*Morus alba* L.
Bisdehydroprotostemonine	101675309	71.562	*Stemona japonica* (Blume) Miq.
Cubebinone	91724200	71.561	*Litsea cubeba* (Lour.) Pers.
9,10-Dihydrophenanthrene-9,10-diol	99889	71.502	*Bletilla striata* (Thunb.) Rchb.f.
Convallasaponin A	441883	71.339	*Polygonatum odoratum* (Mill.) Druce
Yuccagenin	3083608	71.245	*Trigonella foenum-graecum* L.
Methyl (4R)-4-[(5R,10S,13R,14R,17R)-4,4,10,13,14-Pentamethyl-3,7,11,15-tetraoxo-2,5,6,12,16,17-hexahydro-1H-cyclopenta[a]phenanthren-17-yl]pentanoate	21633085	70.88	***Ganoderma lucidum*** **Karst**
Hydnocarpin	5489114	70.561	*Lonicera japonica* Thunb.
Ganoderic acid C2	57396771	70.305	***Ganoderma lucidum*** **Karst**
Kaikasaponin III_qt	188384	70.207	*Abrus precatorius* L.
Trifolirhizin	442827	70.192	*Panax notoginseng* (Burkill) F.H.Chen
			*Pyrrosia lingua* (Thunb.) Farw.
			*Sophora flavescens* Aiton
Cimicifugoside H1	15241163	70.1	***Cimicifuga foetida*** **L**.
27-Hydroxywithanolide B	44576309	70.041	*Datura metel* L.
Kuwanon F	156149	70.023	*Morus alba* L.
MRS2500	44448831	Control	**Synthetic Compounds**

*Bold font indicates the three ideal plants found in this article: licorice, cimicifugae, and ganoderma*.

### Traditional chinese herb analysis

**Figure 3:**
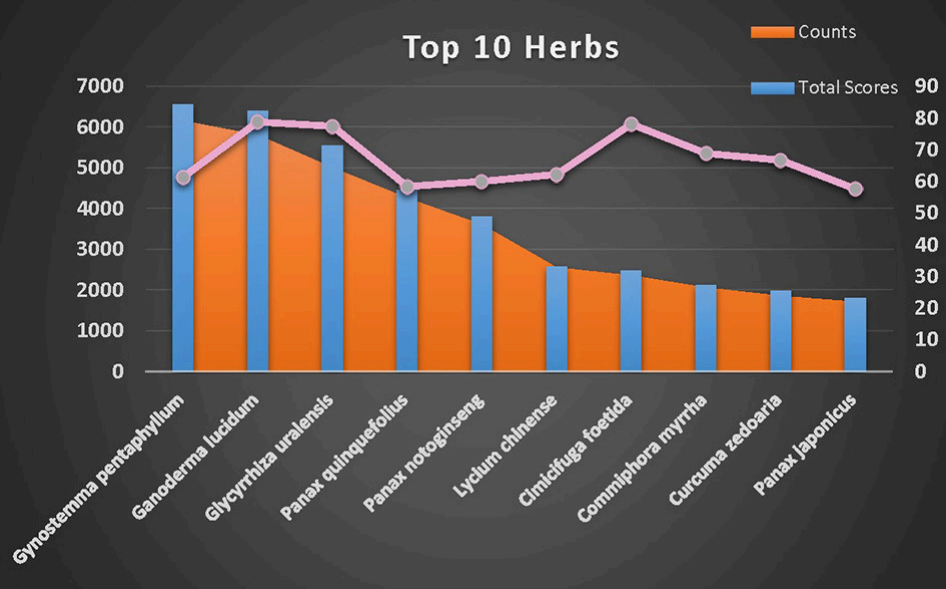
**Top 10 herbs selected by estimating the number of hit compounds (orange area between bars), max DockScore (pink line) and total DockScore (blue bars)**.

Although many TCMs are considered useful for treating CVDs, each herb contains numerous compounds with different P2Y_1_R DockScores. A total DockScore was obtained for each herb by summing the DockScores of its compounds as recorded in the TCMSP (Figure S1). Figure 3 shows the 10 herbs that possessed the highest total DockScores. The IPA indicated that 1890 compounds present in these 10 herbs may be considered drug candidates for hematological diseases and CVDs (Figure S2).

**Figure 4:**
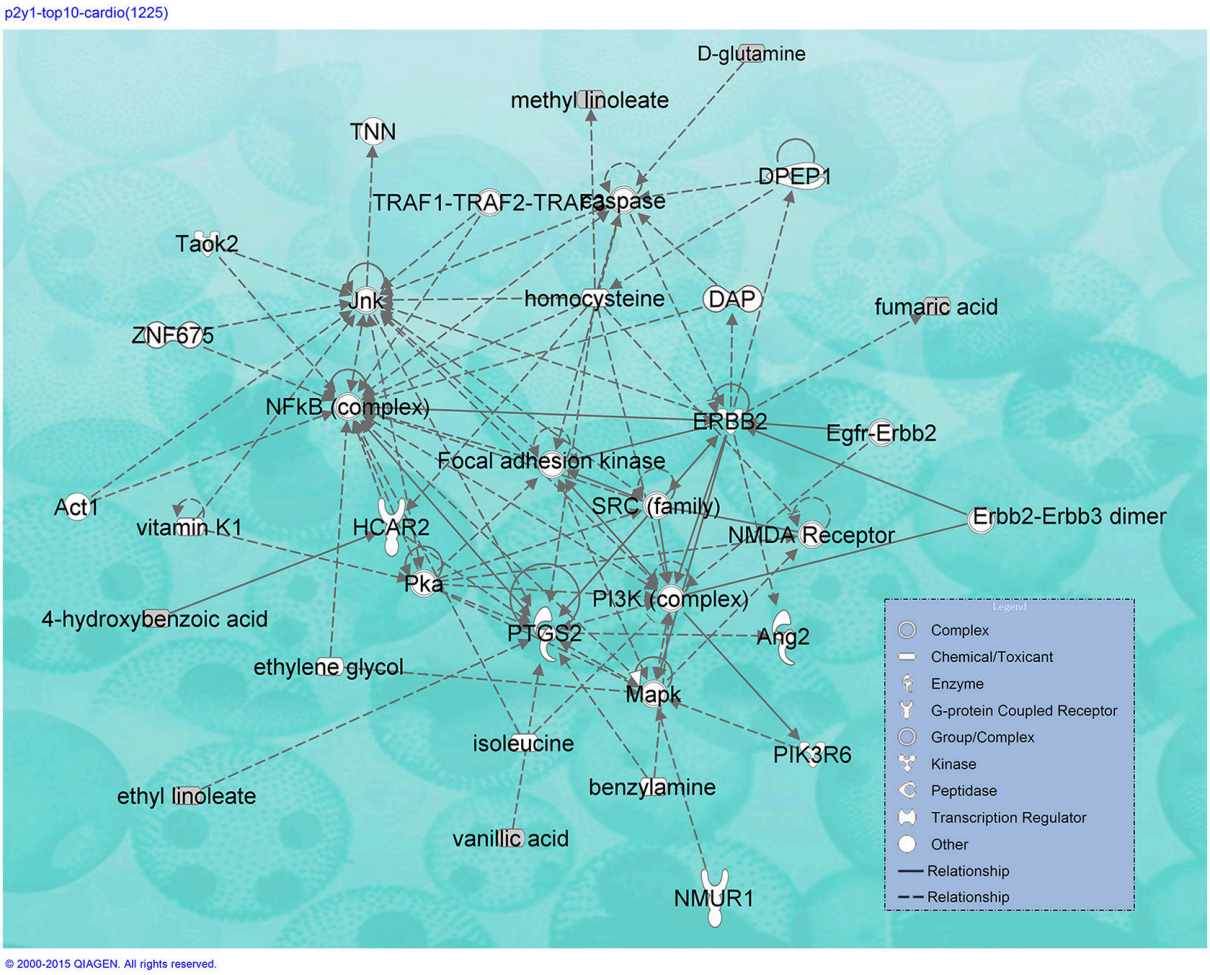
**IPA-mapped CVD pathway for the 1890 compounds distributed in the top 10 herbs**.

Additional GPCRs were mapped by the software. Hydroxycarboxylic acid receptor 2 (HCAR2), a common target for the widely prescribed dyslipidemia drug niacin, is associated with platelet aggregation inhibition (Pike, 2005). In addition, data indicate that the proto-oncogene tyrosine-protein kinase (SRC), phosphoinositide 3-kinase (PI3K), mitogen-activated protein kinase (MAPK), N-methyl-D-aspartate receptor (NMDAR), receptor tyrosine-protein kinase erbB-2 (ERBB2), nuclear factor kappa-light-chain-enhancer of activated B cells (NF-kB), and protein kinase a (Pka) pathways (Figure 4) are activated by the top 10 herbs.

### Candidate compound analysis

Sixteen compounds were associated with platelet aggregation, and all of the relevant herbs were validated in the literature, as shown in Table 2. Among them, ascorbic acid, also known as vitamin C, is a well-known dietary antioxidant that inactivates oxygen free radicals and exerts a variety of protective effects on blood vessels and platelets (Yang et al., 2015). Vitexin and caffeic acid showed significant antiplatelet aggregation activity and were relatively selective inhibitors of platelet aggregation induced by AA and collagen (Afifi and Abu-Dahab, 2012; Lee and Bae, 2015). Rutin, naringin and quercetin, three flavonoid glycosides, were metabolized to phenolic acids via aglycones by human intestinal microflora and have been considered natural prodrugs with anti-platelet activity since the 1990s (Ndhlala et al., 2015). Esculetin is a derivative of coumarin and exists as glycosides and caffeic acid conjugates in *Cimicifugae Rhizoma* (*Cimicifuga foetida* L.) and many other medicinal plants. The systemic usage of esculetin-containing preparations has an anticoagulant effect and may interact with anticoagulant drugs, such as warfarin (Karnewar et al., 2016). Curcumin, a major component of turmeric, is also found in *Cimicifuga foetida* L. and inhibits arachidonate-, adrenaline-, and collagen-induced platelet aggregation. Curcumin inhibited exogenous arachidonate-produced thromboxane B_2_ (TXB_2_) in washed platelets and the incorporation of [^14^C]AA into platelet phospholipids. Moreover, it also inhibited the deacylation of AA-labeled phospholipids (Du et al., 2016). Citric acid and L-malic acid, two main organic acids in *Fructus Choerospondiatis* [*Choerospondias axillaris* (Roxb.) Burtt et Hill] and *Lycii Fructus* (*Lycium chinense* Mill.), possess antioxidant, anti-inflammatory, and antiplatelet aggregation activities (Tang et al., 2013).

**Table 2:** **Compounds related to platelet aggregation extracted from the top 10 herbs**.

	Compounds^*^	**CAS ID**	**Herbs**
1	3,4-Dihydroxyphenylethanol	10597-60-1	***Cimicifuga foetida*** **L**.
2	Caffeic acid	331-39-5	***Cimicifuga foetida*** **L**.
3	Capsaicin	404-86-4	*Panax quinquefolius* L.
4	Cetulinic acid	472-15-1	***Glycyrrhiza uralensis*** **Fisch**
5	Cholesterol	57-88-5	*Commiphora myrrha* (T.Nees) Engl.
6	Chlorogenic acid	327-97-9	*Panax notoginseng* (Burkill) F.H.Chen
7	Citric acid	43136-35-2	*Lycium chinense* Mill.
8	Cscorbic acid	50-81-7	*Lycium chinense* Mill.
9	Curcumin	458-37-7	***Cimicifuga foetida*** **L**.
10	Esculetin	305-01-1	***Cimicifuga foetida*** **L**.
11	Gallic acid	149-91-7	*Gynostemma pentaphyllum* (Thunb.) Makino
			***Glycyrrhiza uralensis*** **Fisch**
			*Panax notoginseng* (Burkill) F.H.Chen
			***Cimicifuga foetida*** **L**.
			*Curcuma zedoaria* (Christm.) Roscoe
12	L-Malic acid	97-67-6	*Lycium chinense* Mill.
13	Naringin	10236-47-2	***Glycyrrhiza uralensis*** **Fisch**
14	Quercetin	117-39-5	*Gynostemma pentaphyllum* (Thunb.) Makino
			*Commiphora myrrha* (T.Nees) Engl.
			*Lycium chinense* Mill.
			***Glycyrrhiza uralensis*** **Fisch**
15	Vitexin	3681-93-4	***Glycyrrhiza uralensis*** **Fisch**
16	Rutin	153-18-4	*Gynostemma pentaphyllum* (Thunb.) Makino
			*Lycium chinense* Mill.
			***Glycyrrhiza uralensis*** **Fisch**

* *Compound information provided by IPA. All compounds had clinical and/or experimental validation determined by IPA chemical database screening. Bold font indicates the three ideal plants found in this article: licorice, cimicifugae, and ganoderma*.

### *Cimicifugae, Ganoderma*, and *Licorice* analysis

According to the dual analyses of results related to both these compounds and plants, we focused on three particular TCM herbs, *cimicifugae* (*Cimicifuga foetida* L.), *ganoderma* (*Ganoderma lucidum* Karst), and *licorice* (*Glycyrrhiza uralensis* Fisch).

*Cimicifugae* (*Cimicifuga foetida* L.) (also known as black cohosh, black snakeroot, and rattlesnake root) has a long tradition of use as a beneficial herbal remedy for joints, muscles, nerve aches, and gynecological disorders in both China and North America. *Cimicifugae* is used as an anti-inflammatory, analgesic and antipyretic remedy in TCM. However, there are few reports of its clinical use in anti-thrombosis. Sodium ferulate (SF) is an active principle compound from *cimicifugae* and has been used in TCM and approved by the State Drug Administration of China for the treatment of cardiovascular and cerebrovascular diseases. SF has antithrombotic, platelet aggregation inhibitory, and antioxidant activities in both animals and humans.

*Ganoderma* (*Ganoderma lucidum* Karst) is a popular medicinal mushroom and has been used in TCM in Asia for the past two millennia to treat numerous diseases. Its regular consumption is believed to preserve human vitality and promote longevity. *Ganoderma* has been used worldwide, and its constituents have been extensively identified; however, the biological potency of this fungus has not yet been sufficiently investigated. According to clinical observations and pharmacologic experiments, *ganoderma* can improve microcirculation in organs and increase the amount of blood. Moreover, in recent years, a series studies focused on *ganoderma* and anti-thrombosis (Kawagishi et al., 1993). The administration of this herb *in vitro* and *in vivo* was found to significantly inhibit ADP-induced platelet aggregation. These inhibitory effects may involve the release of intrinsic ADP from platelets or the metabolites of AA and PAF. Other research showed that purified *ganoderma* fibrinolytic protease protected mice against thrombotic death or paralysis induced by collagen and epinephrine and also activated the partial thromboplastin time (APTT) and thrombin time (TT) in rat platelets (Kumaran et al., 2011). The polysaccharide of *ganoderma* was also found to prolong the blood clotting and bleeding time in rats and decrease the thrombosis and fibrinogen level in blood plasma (Klupp et al., 2016). Evidence indicates that *ganoderma* has potential as an antithrombotic herb because of its antiplatelet activity.

*Licorice* (*Glycyrrhiza uralensis* Fisch; Chinese name Gan-cao) is one of the most indispensable crude drugs in TCM preparations (constituting ~60%), and its wide clinical use widely began in ancient Egyptian, Greek and China in the Second century B.C. *Licorice* comprises the roots of three *Glycyrrhiza* species (*Glycyrrhiza uralensis* Fisch, *Glycyrrhiza glabra* L., and *Glycyrrhiza inflata* Batalin). In traditional clinical treatment, this botanical drug is typically used for allergic-inflammatory disease, gastrointestinal problems, cancer, CVD, and bladder and kidney ailments. Approximately 300 diverse compounds have been isolated from *licorice*, including triterpene saponins and flavonoids, which are responsible for the antiviral, anti-inflammatory, antitumor, antitussive, anti-oxidant, antispasmodic, and metabolic syndrome preventive activities. However, evidence suggests that licorice has anti-thrombosis activity and can be used to treat CVDs.

**Figure 5:**
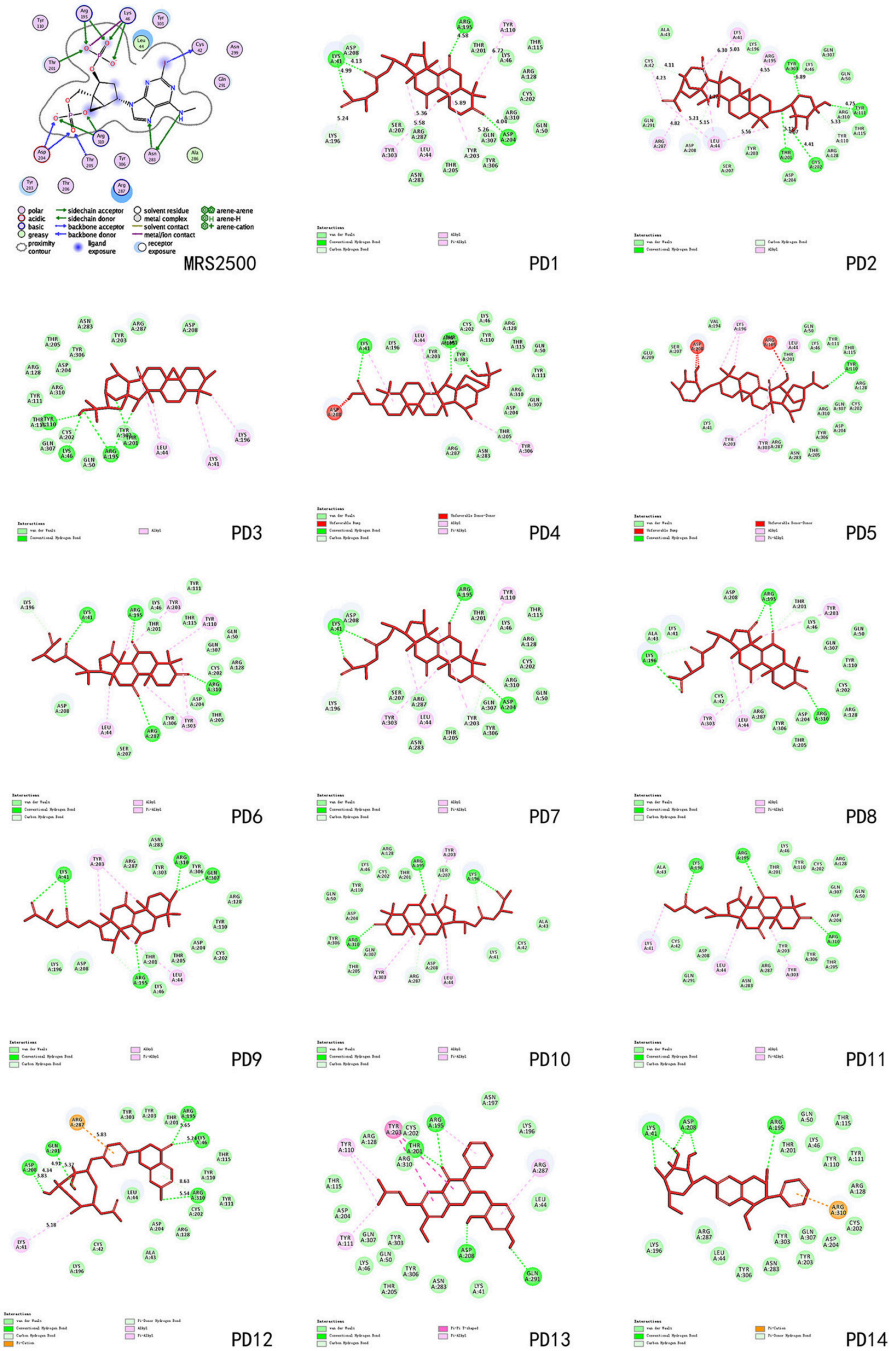
**Comparison of 14 compounds in the binding model structure with MRS2500**.

These three herbs contained 14 compounds with a DockScore above 70 (Table 3). By calculating the binding energies between P2Y_1_R and these 14 compounds, the binding efficiency and loss of conformational entropy and energy of a bound ligand can be obtained. These compounds have different ranges of ligand energies, Van der Waals energies and bond energies compared with the ligand MRS2500. However, according to CHARMm command-based calculations, all 14 have similar rotational and translational entropy values, indicating that these compounds all have the same effective binding efficiency as the original ligand MRS2500 (Figure 5).

**Table 3:** **Chemical information of candidate compounds from *Cimicifuga foetida* L., *Ganoderma lucidum* Karst, *and Glycyrrhiza uralensis* Fisch**.

**Herbs**	**ID**	**Name**	MW^*^	**AlogP**	**H-d**		**H-a**	**RB**	**NR**	**DL**	**CYP2D6 Pre**		**HPT Pre**		**PPB Pre**	
*Cimicifuga foetida* L.	PD-1	methylcimicifugoside_qt	556.81	3.21	1		7	6	4	0.24	−0.579352	false	0.729381	true	2.26562	true
	PD-2	cimiside e	602.89	3.32	4		8	5	4	0.16	−3.41278	false	−4.76741	false	−13.1301	false
	PD-3	cimigenol	488.78	3.53	3		5	5	4	0.40	−2.4378	false	−2.71708	true	−4.66017	false
	PD-4	cimicifoetiside a_qt	532.79	4.30	3		7	6	4	0.33	−5.61872	false	−3.45153	true	−2.03733	true
	PD-5	7,8-didehydrocimigenol	618.89	2.05	5		9	6	4	0.15	−3.64524	false	−3.68623	true	1.14555	true
*Ganoderma lucidum* Karst	PD-6	ganodermic acid O	528.70	1.17	2		8	6	4	0.79	−2.62642	false	−3.11195	true	0.987057	true
	PD-7	ganoderic acid J	514.72	2.29	2		7	6	4	0.81	−4.73633	false	−6.3584	false	−0.353049	true
	PD-8	ganoderic acid E	512.70	2.25	1		7	6	4	0.81	−4.23523	false	−4.08921	true	−0.00661775	true
	PD-9	ganoderic acid C2	518.76	2.37	4		7	5	4	0.81	−3.1875	false	−9.81044	false	1.39596	true
	PD-10	(2S,6R)-6-[(5R,7S,10S,13R,14R,17R)-7-hydroxy-4,4,10,13,14-pentamethyl-3,11,15-trioxo-1,2,5,6,7,12,16,17-octahydrocyclopenta[a]phenanthren-17-yl]-2-methyl-4-oxoheptanoic acid	514.72	2.29	2		7	3	8	0.81	−9.70253	false	−12.8998	false	−4.17637	false
	PD-11	methyl (4R)-4-[(5R,10S,13R,14R,17R)-4,4,10,13,14-pentamethyl-3,7,11,15-tetraoxo-2,5,6,12,16,17-hexahydro-1H-cyclopenta[a]phenanthren-17-yl]pentanoate	470.66	2.69	0		6	3	8	0.81	−9.06642	false	−9.62875	false	−2.87221	false
*Glycyrrhiza uralensis* Fisch	PD-12	6″-O-acetylliquiritin	444.47	2.33	3		9	1	7	0.82	−6.31325	false	−6.56771	false	−1.12272	true
	PD-13	glyasperin E	444.51	6.41	2		6	3	7	0.75	−7.23522	false	−7.38604	false	−1.11392	true
	PD-14	2′,7-Dihydroxy-4′-methoxyisoflavan-7-O-β-d-glucopyranoside.	434.48	0.59	5		9	3	8	0.73	−9.10824	false	−8.00786	false	−3.38568	false

* *MW, molecular weight; AlogP, log of the octanol-water partition coefficient according to Ghose and Crippen's method; H-d, H-donors; H-a, H-acceptors; RB, number of rotatable bonds, defined as single bonds between heavy atoms that are both not in a ring and not terminal, i.e., connected to a heavy atom that is attached to only hydrogens. As a special case, amide C-N bonds are not rotatable; NR, base rings, defined as the number of rings in the smallest set of smallest rings (SSSR); DL, drug-likeness index; CYP2D6P, cytochrome P450 2D6 prediction; HPT Pre, hepatotoxic prediction; PPB Pre, plasma protein binding capability prediction*.

A drug-likeness (DL) analysis was performed to determine whether these compounds have potential uses as drugs. The DL index values of these compounds were calculated using the Tanimoto coefficient and are presented in the TCMSP database. A molecule with DL > 0.18 was considered a drug-like compound and was selected for the following processes. Cytochrome P450 2D6 (CYP2D6) is involved in the metabolism of a wide range of substrates in the liver, and its inhibition is related to most drug-drug interactions. The CYP2D6 model can predict candidate compounds for CYP2D6 enzyme inhibition; therefore, investigating CYP2D6 inhibition is a required part of the regulatory procedures involved in the drug discovery and development process. Discovery Studio ADME/T Descriptors contain a computational model for compounds inhibiting the CYP2D6 enzyme, which was developed from a training set of 151 structurally diverse compounds with known CYP2D6 inhibition constants. This experiment was performed with modified Bayesian learning, and all 14 compounds scored below the cutoff Bayesian score of 0.161 and were not classified as CYP2D6 inhibitors. Therefore, these compounds are unlikely to have drug-drug interactions and can improve the efficiency and safety of drugs, which has significant clinical importance. The hepatotoxicity model can predict potential organ toxicity and was developed from the available literature data for 436 compounds known to exhibit liver toxicity or trigger dose-related elevated aminotransferase levels in more than 10% of the human population. For these 14 compounds, eight had no hepatotoxic activity, including four compounds from licorice (Table 4).

**Table 4:** **P2Y_1_R binding energies for compounds PD-1~PD-14**.

**Compound**	**Ligand energy**	**Improper energy**	**Angle energy**	**Dihedral energy**	**Electrostatic energy**	**Van der Waals energy**	**RMS gradient**	**Bond energy**	**Rotational entropy**	**Translational entropy**	Ligand entropic energy^*^
MRS2500	112.642	0.01780	37.4877	18.4862	31.3132	4.02	27.0966	21.3167	38.0197	32.3073	−20.968
PD-1	132.048	0.08685	53.3583	115.673	−34.8348	−21.7701	14.3482	19.5344	38.0119	32.3052	−20.965
PD-2	158.233	0.00162	40.7545	118.223	−0.04683	−23.6102	8.53944	22.9105	38.5017	32.5422	−21.1817
PD-3	173.499	0.00000	95.1922	83.3181	−11.2569	−14.6189	8.2638	20.864	37.2045	31.9167	−20.6085
PD-4	137.537	0.00781	35.496	85.7151	−38.3119	35.2412	25.7248	19.3887	37.7349	32.1738	−20.8433
PD-5	326.639	0.60045	50.483	136.935	27.8459	90.152	67.7923	20.6223	38.8614	32.6203	−21.3123
PD-6	80.7362	0.13864	26.5836	75.593	−35.1109	0.98494	17.0155	12.547	37.7973	32.1509	−20.8551
PD-7	85.0095	0.13282	30.6886	85.335	−32.4446	−10.3341	15.0403	11.6317	37.2454	31.8042	−20.5871
PD-8	104.733	0.06613	31.4111	89.5891	−38.5655	9.27464	20.1861	12.9572	37.6091	32.071	−20.7751
PD-9	72.005	0.10815	26.9039	75.2766	−34.1692	−8.48898	16.572	12.3746	37.9219	32.0593	−20.8649
PD-10	123.831	0.11197	31.7549	102.652	−40.3419	15.6907	18.6129	13.9629	38.125	32.0942	−20.9359
PD-11	77.6839	0.07226	27.2305	82.7531	−33.2483	−11.1572	14.5606	12.0335	37.7967	32.071	−20.8311
PD-12	51.0403	0.02020	13.3322	38.6903	−31.9949	−5.449	27.1373	36.4415	37.2514	31.6337	−20.5381
PD-13	113.581	0.00089	13.4249	43.8391	16.1906	4.25157	21.2566	35.8735	37.303	31.5659	−20.5333
PD-14	100.668	0.03287	8.12721	44.1692	8.49017	−6.09511	25.1593	45.9435	37.5527	31.634	−20.628

* *Ligand entropic energy, rotational and translational entropy of the ligand (kcal/mol); rotational entropy, rotational entropy calculated from the principal moments of inertia for the ligand (kcal/mol); translational entropy, corrected translational entropy for the ligand (kcal/mol)*.

The scaffold and pharmacophore are two basic elements of drug molecules. The pharmacophore consists of discrete atoms, groups, and fragments responsible for the physic-chemical features, and their arrangement controls the specific activities. However, the pharmacophore must incorporate an integrated scaffold to form an actual molecule, and thus, implanting the same pharmacophore on various scaffolds results in structurally diverse compounds that act on the same biological target. The promiscuity of receptors indicates that scaffolds can be rationally transformed based on the flexibility and plasticity of the receptors, thereby imbuing receptor binding sites with diversity and variability. Compared to traditional high-throughput screening methods, the scaffold-based approach can facilitate higher initial synthetic efforts guided by the co-crystal structure.

**Figure 6:**
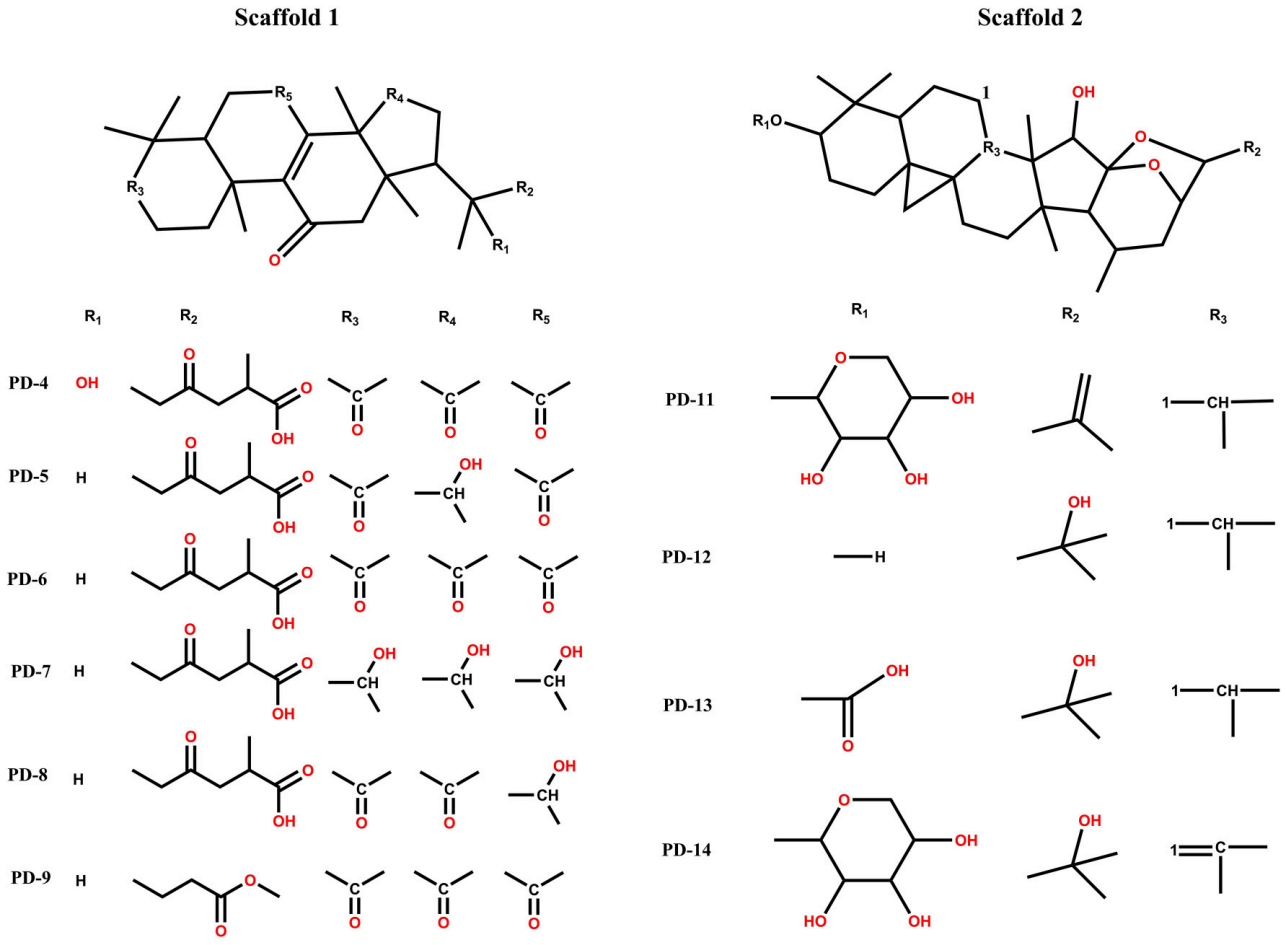
**Two types of scaffolds in *Cimicifuga foetida* L., *Ganoderma lucidum* Karst, and *Glycyrrhiza uralensis* Fisch**.

Among these 14 compounds, two triterpene scaffolds are found in 10 compounds. Scaffold 1 was found in compounds PD-4, 5, 6, 7, 8, and 9, whereas scaffold 2 was found in compounds PD-11, 12, 13, and 14 (Figure 6). These two scaffolds could be further developed into more potent and selective drug-like ligands by an iterative process including co-crystallography of the complex constructed by candidate compounds with the P2Y_1_R protein. These candidate compounds could subsequently be used for chemical syntheses followed by structure-guided computational design. An ideal DL scaffold should not only form key interactions with the protein target but also possess a conserved binding mode that can tolerate small substitutions.

## Conclusion

Platelet aggregation occurs when platelets adhere to each other at sites of vascular injury. This process has long been recognized as critical for hemostatic plug formation and thrombosis, which is the final product of the aggregated platelets that formed a platelet plug. Vitamin K antagonists and warfarin are often taken orally to reduce thromboembolic occurrence; however, both of these drugs have an unwanted side effect of an increased risk of bleeding.

Uncontrolled platelet aggregation is critical in thrombosis, which may cause heart attacks, unstable angina and other systematic disorders. The inhibition of platelet aggregation is fundamentally important in the treatment and prevention of CVDs. Platelet aggregation can regulated by G protein-coupled P2Y receptor families, particularly P2Y_12_ and P2Y_1_ (Aslam et al., 2013). P2Y_1_ is required for platelet shape change in response to ADP and is also a principal receptor in mediating both physiological and pathological ADP-induced platelet aggregation. At higher ADP concentrations, P2Y_1_-deficient platelets become partially aggregated. *In vivo*, the bleeding time was increased by the lack of P2Y_1_ expression, which could protect against collagen- and ADP-induced thrombosis. It is sufficient to block ADP-induced platelet aggregation by utilize specific antagonists inhibit the signaling through P2Y1 receptor (Jin and Kunapuli, 1998).

MRS2500 is a new antiplatelet drug that has strong antithrombotic activity in systemic thromboembolism induced by adrenaline infusion. In P2Y_1_R, the antagonist MRS2500 may prevent the movement of P2Y_1_R helix structures and stabilize the receptor in an inactive state. The nucleotide-like antagonist MRS2500 of P2Y_1_R has numerous anchor points on different receptor domains in hydrophilic and charged sites. The interactions of both P2Y_1_ and P2Y_12_R with their nucleotide ligands reveal disparate binding modes between these two P2YR subfamilies, broadening our understanding of the diversity of signal-recognition mechanisms in GPCRs and providing additional potential ligands and diversity binding models to regulate the proteins of these subfamilies. MRS2500 has is an ideal antagonist of P2Y_1_R that can prevent platelet aggregation caused by thrombosis.

The pharmacological actions of TCMs activate blood and resolve stasis to treat thrombosis. Many Traditional Chinese herbs and Chinese patent medicines are widely applied in clinical use to treat thrombosis and have good prospects in Chinese Medicine. These drugs are used for promoting blood circulation for removing blood stasis (PBCRBS) and are involved in the clinical treatment of cardiovascular-related diseases, particularly as anti-thrombosis agents. Compared to Western medicines, TCM herbs have advantages of mild action, multiple pathways, multiple targets, and fewer adverse reactions. According to the network pharmacology analysis of PBCRBS herbs, the most frequently used TCMs contain *Danshen* (*Salvia miltiorrhiza* Bge.), *Chuanxiong* (*Ligusticum chuanxiong* hort), *Honghua* (*Carthamus tinctorius* L.), *Sanqi* (*Panax notoginseng* Burk), and *Danggui* [*Angelica sinensis* (Oliv.) Diels]. However, analyzing these herbs and their compounds revealed that they mainly exerted anti-thrombosis activity by affecting the nitric oxide synthase 2 (NOS2), prostaglandin-endoperoxide synthase 2 (PTGS2), tumor necrosis factor (TNF), and interleukin 1 beta (IL-1β) pathways. Currently, no research into the application of these herbs/compounds to P2Y_1_ receptors is available. Because the construction of MRS2500 and the combination model between MRS2500 and P2Y_1_R has been established, numerous TCMs can be utilized to treat thrombosis in a novel and mild manner. A combination of antiplatelet dugs is more efficient than a single drug at inhibiting the multiple pathways of platelet activation. Furthermore, natural compounds extracted from herbs have fewer side effects than synthetic compounds. We investigated the herbs and their compounds with anti-platelet aggregation activities similar to those of MRS2500.

Overall, in this study, a total of 8987 compounds from TCMs filtered by Lipinski's rule of five were predicted have ideal oral-intake properties. Among them, 1656 compounds distributed in 443 herbs were able to dock into the P2Y_1_R-MRS2500 structure in 16,317 poses. A total of 38 compounds had a DockScore above 70.0 and may have significant potential for development into anti-thrombosis drugs. We also investigated *Cimicifuga foetida* L., *Ganoderma lucidum* Karst and *Glycyrrhiza uralensis* Fisch. These herbs and their chemical constituents have not been widely used in anti-thrombosis treatment and may have potential as new drugs for the inhibition of platelet aggregation.

## Author contributions

HL and PX designed the experiment. FY performed the experimental computing, data analysis, figure drawing, and paper writing. LS, YP, CH, and LX collected the compound information.

### Conflict of interest statement

The authors declare that the research was conducted in the absence of any commercial or financial relationships that could be construed as a potential conflict of interest.

## Supplementary material

Supplementary Data Sheet 1 **Total original DockScores of compounds collected from TCMSP**.

Figure S1 **Total plants DockScore results analysis**. (Blue columns represent total DockScore and Orange polyline represents the tendency of the highest DockScore of each plant).

Figure S2 **Canonical pathway analysis calculated in Ingenuity Pathway Analysis (IPA)**.
