# Supplementary figures and images for: In silico Approach for Anti-Thrombosis Drug Discovery: P2Y1R Structure-Based TCMs Screening
Source: Front Pharmacol. 2017 Jan 9;7:531. doi: 10.3389/fphar.2016.00531 (PMC5220089; doi:10.3389/fphar.2016.00531)

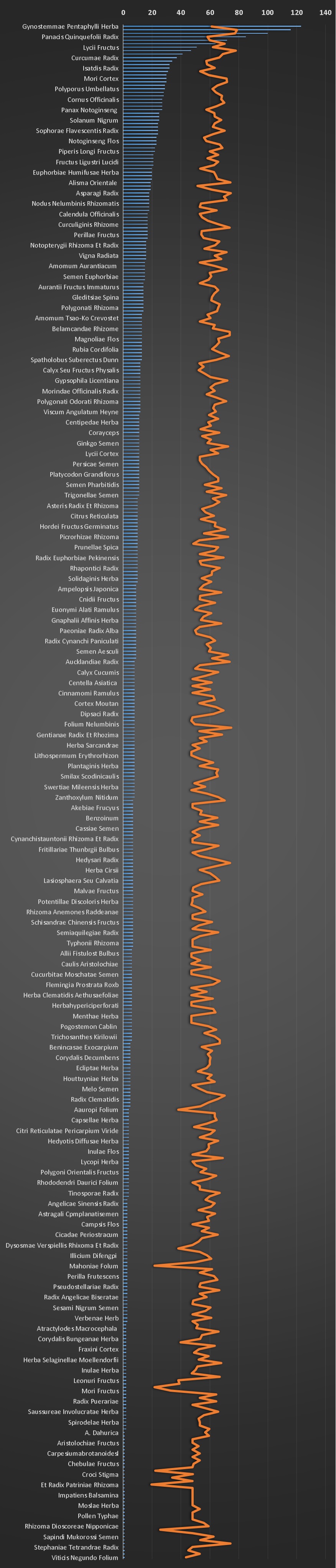

Supplement: Figure S1 — Total plants DockScore results analysis. (Blue columns represent total DockScore and Orange polyline represents the tendency of the highest DockScore of each plant). [file Image1.JPEG]

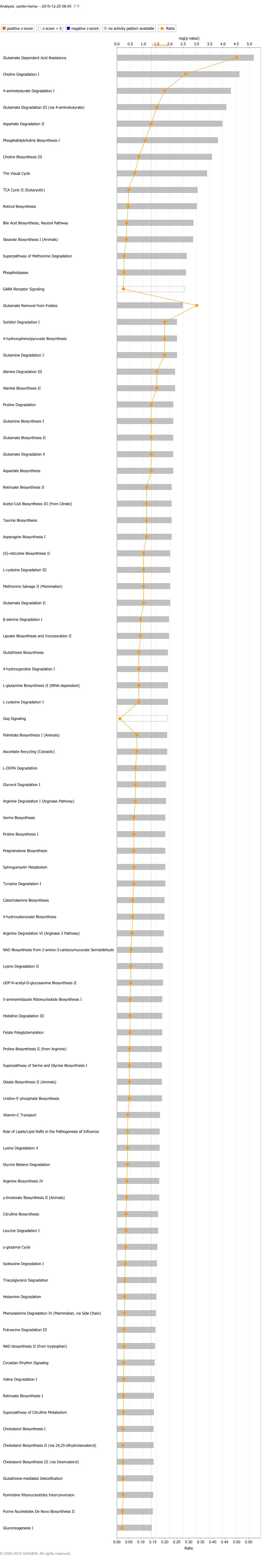

Supplement: Figure S2 — Canonical pathway analysis calculated in Ingenuity Pathway Analysis (IPA). [file Image2.JPEG]
